# Effects of Installing Different Types of Cooling Fins on the Cold Side of a Thermoelectric Power Generation Device on the Thermal Efficiency and Exergy Efficiency of Power Cable Surface Waste Heat Recovery

**DOI:** 10.3390/mi14081591

**Published:** 2023-08-13

**Authors:** Zihao Hu, Francisco de León, Rizhou Wang, Yanzhe Li

**Affiliations:** Department of Electrical and Computer Engineering, Tandon School of Engineering, New York University, New York, NY 11201, USA; zh1435@nyu.edu (Z.H.); rw3157@nyu.edu (R.W.)

**Keywords:** thermoelectric power generation device, power cable, cooling fins, thermal efficiency, exergy efficiency

## Abstract

This study investigates the thermal efficiency and exergy efficiency of a thermoelectric power generation device for recovering power cable surface waste heat. Numerical simulations are conducted to analyze the impact of different types of cooling fins on the system’s performance. The results demonstrate that the installation of cooling fins improves heat transfer efficiency and enhances the thermoelectric power generation device’s output power. Among the various fin designs, the system equipped with cooling fins with 17 teeth exhibits the highest performance. These findings highlight the importance of fin design in optimizing the system’s thermal efficiency and exergy efficiency. This study provides valuable insights for the development and improvement of thermoelectric power generation systems for power cable surface waste heat recovery applications.

## 1. Introduction

With the continuously growing energy demand and increase in environmental issues, the scientific community has intensified their research and development efforts with respect to renewable energy technologies. Thermoelectric power generation, a renewable energy technology that converts natural temperature differences into electrical energy, has gained considerable attention. On one hand, power cables are essential components of the power system for long-distance electricity transmission. On the other hand, conventional transmission cables release thermal energy into the environment during energy transmission, leading to reduced energy utilization efficiency. To address this issue, researchers have explored integrating thermoelectric power generation technology with power cables to recover the heat generated during cable operation and convert it into usable electrical energy. This combined solution has the potential to significantly enhance secondary energy utilization efficiency, improve energy recovery efficiency, reduce energy waste in power systems, and contribute to the sustainable development of the power transmission industry. Thermoelectric power generation technology operates based on the Seebeck effect of semiconductor thermoelectric materials, generating electrical energy when there is a temperature difference across the material. Although the technology has been successfully applied for small-scale energy recovery, its application in large-scale power cables is still limited. By integrating thermoelectric modules into the cable surface and utilizing the temperature difference between the cable surface and the environment, the waste heat generated during cable operation can be efficiently converted into electrical energy, improving the overall energy utilization efficiency and reducing our reliance on traditional energy sources.

Thermoelectric power generation technology offers significant advantages, such as the efficient utilization of low-quality thermal energy sources, environmentally friendly operation, and applicability in various sectors. Continued research and innovation in this field can lead to cleaner and more sustainable energy solutions, reducing our reliance on traditional energy sources and addressing environmental challenges. Zhao et al. demonstrated the potential of thermoelectric generators in capturing energy from waste heat in wet flue gas recovery research. This technology improves energy utilization efficiency and reduces energy wastage [1]. Their further research on thermoelectric generators with heat pipes showcased the effectiveness of thermoelectric power generation in waste heat recovery, providing a promising approach for efficient energy conversion [2]. Additionally, their study on waste gas thermoelectric generator systems utilizing heat-transfer-fluid circulation analyzed the impact of fluid circulation on the system’s performance. The research results demonstrated the potential of using heat-transfer-fluid circulation to enhance energy recovery efficiency in waste gas thermoelectric generator systems [3]. Fu et al. [4] conducted experiments on a vehicle-mounted thermoelectric generator (TEG) used in a cold chain logistics transportation vehicle. They evaluated the TEG’s working efficiency and exergy efficiency under various conditions. The results showed that the TEG exhibited promising performance in converting waste heat into usable electrical energy in cold chain logistics transportation vehicles. Li et al. [5,6,7] conducted a study on the impact of filling the core flow region of a thermoelectric generator with a porous foam metal on the performance of the thermoelectric generator (TEG). They improved the experimental setup by using thermoelectric modules with different characteristics and optimizing the heat transfer in the core flow region. The results showed that the choice of thermoelectric modules had a significant influence on the TEG’s performance, and the optimization of heat transfer in the core flow region positively affected the TEG’s power generation characteristics. He et al. [8,9] studied the impacts of different cooling methods on a thermoelectric generator used for engine waste heat recovery. They proposed an optimization design method based on exhaust gas parameters to maximize power output and efficiency. The results provided insights for selecting suitable cooling methods and optimizing design parameters for efficient energy conversion. Zhao et al. conducted numerical studies and performance analyses on various thermoelectric power generation devices. They investigated waste gas thermoelectric generators with perforated plates [10], automotive waste gas thermoelectric generator systems with an intermediate fluid [11], and intermediate-fluid thermoelectric generators used for automotive waste heat recovery [12]. The research results revealed that the optimized thermoelectric generators exhibited superior performance in capturing and converting waste heat from automotive exhaust, leading to enhanced energy efficiency and waste heat recovery efficiency in automotive applications. Ge et al. [13] optimized the structure of the thermoelectric modules in a photovoltaic–thermoelectric hybrid concentration system to enhance the system’s overall performance and efficiency. They also conducted experiments on thermoelectric generators with different numbers of modules for waste heat recovery. The study compared the TEGs’ performances with varying module numbers and analyzed the optimal configuration for the desired power generation characteristics [14]. Luo et al. [15] presented a comprehensive hybrid transient computational fluid dynamics and thermal resistance model for automobile thermoelectric generators. The model combined CFD simulations with a thermal resistance network to accurately predict the temperature distribution and heat transfer within the ATEG. The study enhanced the understanding of and capability to model ATEGs for improved design and performance optimization. Li et al. [16,17,18] examined the effect of enhancing the core flow heat transfer on the power generation characteristics of thermoelectric generators with different performances. They analyzed the impact of improving the heat transfer in the core flow region on the overall power generation of the TEGs. The study emphasized the importance of efficient heat transfer in enhancing the performance of a TEG.

Among the various methods of enhancing the performance of a thermoelectric power generator, the addition of fins is a convenient and effective approach. Fins represent a common heat conduction enhancement element that can increase the efficiency of the heat transfer between the thermoelectric generator and the heat source, thereby improving the energy conversion efficiency. By adding fins, the surface area of the TEG is increased, allowing it to better absorb the heat released by the heat source and convert it into electrical energy. This increases the power generation capacity of the TEG while reducing energy waste and improving energy utilization efficiency. Therefore, adding fins is a simple and effective method that can be used in various applications to enhance the performance of a thermoelectric power generator. Wang et al. [19] focused on improving the performance of a thermoelectric generator through the design of a heat sink using a two-stage optimization approach. The study improved the energy conversion efficiency of thermoelectric generators by optimizing the design of heat sinks. Liu et al. [20] investigated the performance of a thermoelectric generator system used for waste heat recovery in the bronze-ingot-casting industry. They utilized a plate-fin heat sink to enhance the system’s efficiency at converting waste heat into electricity. Seo et al. [21] conducted a numerical study on the performances of thermoelectric modules with differently shaped heat sinks. The study was conducted by analyzing the effects of various heat sink designs on the overall performance of the thermoelectric modules. Pujol et al. [22] carried out a design optimization for a plate-fin heat sink with forced convection for a single-module thermoelectric generator. The research results showed that the optimization had effectively improved the efficiency and power generation capabilities of the thermoelectric generator. Kang et al. [23] explored the energy impact of a heat-pipe-assisted microencapsulated-phase-change-material heat sink for a hybrid panel consisting of photovoltaic and thermoelectric generators. The study showed that the overall energy conversion efficiency of a hybrid panel can be improved by incorporating heat pipe technology and phase change materials into the design of the heat sink. Zheng et al. [24] proposed a passive evaporative-cooling heat sink method to enhance the low-grade waste heat recovery capacity of thermoelectric generators. The study developed an efficient cooling method to improve the energy conversion efficiency of thermoelectric generators. Lee [25] presented a comprehensive book on thermal design, covering various topics such as heat sinks, thermoelectrics, and heat pipes. This book provides valuable insights into the design of efficient thermal systems for heat transfer, such as thermoelectric conversion and finned heat sinks. Jang et al. [26] conducted a 3D numerical simulation and experimental comparison of the turbulent flow of venting flue gas using thermoelectric generator modules and a plate-fin heat sink. The study optimized the heat transfer and improved the energy conversion efficiency of the system by enhancing the flow intensity of the hydrodynamics in the thermoelectric system. Ma et al. [27] conducted a numerical study on the thermoelectric–hydraulic performance of a thermoelectric power generator with a plate-fin heat exchanger featuring longitudinal vortex generators. The study optimized the heat exchanger’s design to enhance the system’s heat transfer and energy conversion efficiency. Naphon et al. [28] investigated liquid cooling in a mini-rectangular-fin heat sink with and without thermoelectric elements for CPU cooling. The study evaluated the cooling performance of the heat sink and its effectiveness in dissipating heat from electronic devices. Wang et al. [29] focused on optimizing the fin distribution in a heat exchanger for a thermoelectric generator to improve temperature uniformity. The study enhanced the system’s thermal performance and overall efficiency. Chen et al. [30] explored power generation using a thermoelectric generator with plate fins for recovering low-temperature waste heat. The study developed an efficient waste heat recovery system to produce electricity from low-grade heat sources. Wang et al. [31] conducted a performance evaluation of an automotive thermoelectric generator with a non-isometric distributed-fin heat exchanger. The study assessed the efficiency and practicality of the thermoelectric generator in automotive waste heat recovery applications.

By combining power cables with thermoelectric power generation technology, it is possible to achieve the green, environmentally friendly, and efficient secondary utilization of renewable energy. This innovative approach involves recovering the waste heat generated on the surfaces of transmission power cables during operation and converting it into electrical energy, thereby improving energy utilization efficiency. In further enhancing the efficiency of recovering waste heat from cables, incorporating heat sink fins plays a crucial role. Heat sink fins are common thermal conduction enhancement elements that can increase the heat transfer efficiency between the thermoelectric power generation device and the heat source. By optimizing the design and layout of the heat sink fins, the surface area of the device can be increased, allowing it to better absorb the heat released by the cable surface waste heat. Therefore, this study investigated the integration of heat sink fins into the surfaces of the cables in a waste heat recovery thermoelectric power generation system. The research results show that this method improves the energy conversion efficiency, increases the device’s output power, and reduces energy wastage. Additionally, the study comprehensively evaluated the performance of the power-cable-surface-waste-heat-recovery thermoelectric power generation device from different perspectives, using thermal efficiency and exergy efficiency as two assessment criteria.

## 2. Methodology

### 2.1. Model Structure

**Figure 1:**
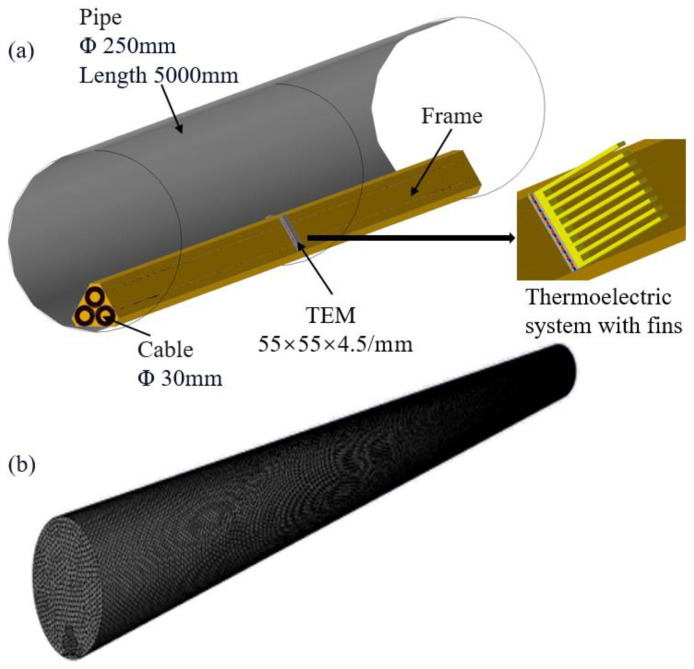
(**a**) Structure of the system; (**b**) mesh division of the system.

Figure 1a illustrates the structures of a thermoelectric power generation device for recovering waste heat from cable surfaces and the thermoelectric power generation system with fins. The device consists of the following components: a 5000 mm long, 250 mm diameter pipe; three 30mm diameter cables arranged in a clover-leaf shape; a frame; and two thermoelectric modules. To facilitate the installation of the thermoelectric modules and heat conduction, the cable exteriors are encased in a copper frame. In this device, the air velocity at the inlet of the pipe is set at 5 m/s, with a temperature of 20 °C. The temperature on the cable surface is set to a constant 90 °C. For observation and analysis purposes, the thermoelectric modules are only installed in the middle section of the cables. The dimensions of the thermoelectric modules (TEM) are 55 × 55 × 4.5 mm. Compared to the thermoelectric generator models described in References [6,18], the model proposed in this study demonstrates a significant reduction in size and is approximately 4 and 8 times smaller than the other models, respectively. This substantial decrease in size has successfully enabled the miniaturization of thermoelectric power generation devices. Small-scale thermoelectric generators offer several advantages, including enhanced space efficiency, improved portability, a faster thermal response time, enhanced heat transfer efficiency, and cost savings in fabrication processes. These advantages contribute to the widespread adoption and versatility of miniaturized thermoelectric generators across diverse applications. Figure 1b depicts the mesh division of the heat-recovery thermoelectric power generation device for cable-surface waste heat. To validate the accuracy of the simulation results, five different mesh quantities were selected. Through validation, a model with a mesh quantity of 1,051,050 was chosen as the standard for subsequent calculations. To further validate the accuracy of our numerical simulations, we conducted a comparison of our simulation results with those presented in Reference [32]. Upon comparing the Nusselt number (*N*u) values with those reported in the reference, we found a discrepancy of 6%. Considering the existing literature, this level of error in our simulated *N*u values falls within an acceptable range. This comparison provides substantial evidence for the reliability of our computational model and underscores the accuracy of our simulation results.

In this study, we employed a comprehensive fluid–solid–electric coupled model which operates under the assumption of a steady-state condition. The model encompasses a three-dimensional representation of the fluid, which is characterized by an incompressible turbulent flow. The material properties within the model are assumed to be isotropic, allowing for a simplified yet representative depiction of the system. It is important to note that certain aspects are not accounted for in the computational process. Specifically, we did not consider contact thermal resistance and radiation effects within the scope of this study. While these factors can influence the thermal behavior of the system, their omission enabled us to focus on the core interactions of fluid dynamics, solid conduction, and electrical phenomena. This approach facilitates a focused exploration of the interplay between these primary variables and their implications for the device’s performance.

### 2.2. Model Settings

#### 2.2.1. Turbulence Governing Equations

The mass, momentum, and energy conservation equations are as follows:

(1) $$\nabla \cdot \vec{v}=0$$

(2) $$\nabla \cdot \left(\vec{v}\vec{\nu }\right)=-\frac{1}{\rho }\nabla_{p}+\nabla \cdot \left(\mu \nabla \vec{v}\right)$$

(3) $$\rho c\vec{v}\cdot \nabla T=\nabla \cdot \left(\gamma \nabla T\right)$$

$\mathrm{where}\;\vec{v}—$velocity vector, $\nabla_{p}—$steady-state pressure, T—temperature, ρ—density, μ—kinetic viscosity, and c—specific heat capacity.

#### 2.2.2. Thermoelectric-Module-Governing Equations

The solid heat transfer equations are as follows:

(4) $$\lambda_{s}\nabla^{2}T=0$$

(5) $$\nabla \cdot \left(\lambda_{p,n,copper}\nabla T_{p,n,copper}\right)+{\dot{S}}_{T}=0$$

(6) $$\nabla \cdot \left(\lambda_{ce}\nabla T\right)=0$$

(7) $${\dot{S}}_{T}=\{\begin{array}{c} \frac{1}{\sigma_{\gamma }}J_{p}^{2}-\nabla \alpha_{p}T_{p}\vec{J_{p}};\\ \frac{1}{\sigma }J_{n}^{2}-\nabla \alpha_{n}T_{n}\vec{J_{n}};\\ \frac{1}{\sigma_{copper}}{\vec{J}}_{copper}^{2}; \end{array}$$

where $\lambda_{s}$—solid wall thermal conductivity, *λ_p,n,copper_*—*p*-leg, *n*-leg, and copper thermal conductivity, *λ_ce_*—ceramic plate thermal conductivity, *T_p,n,copper_*—*p*-leg, *n*-leg, and copper temperature, *S_T_*—source of energy, *J*—current density, and *σ*—electrical conductivity.

The numerical simulation software used in this study was ANSYS Fluent 2021 R2. The heat transfer process was computed using the fluid–thermal coupling approach. In this study, the SIMPLEC algorithm was employed, and a second-order upwind scheme was used to solve for the momentum and dissipation rate.

### 2.3. Thermal Efficiency and Exergy Efficiency of the System

Typically, the performance evaluation of a heat-recovery thermoelectric power generation device uses thermal efficiency as a reference criterion. However, in this study, an additional evaluation metric called exergy efficiency was introduced to assess the performance of the cable-surface-waste-heat-recovery thermoelectric power generation device model. These two kinds of evaluation metrics provide a comprehensive assessment of the performance of the thermoelectric power generation device from different perspectives. Thermal efficiency primarily focuses on the overall energy conversion efficiency and measures the proportion of heat energy converted into electrical energy by the thermoelectric power generation device. It reflects the efficiency and conversion effectiveness of energy utilization and provides a direct evaluation of the device’s performance in energy conversion. On the other hand, exergy efficiency emphasizes the utilization efficiency of the available temperature-difference resources by the thermoelectric power generation device. It considers the relationship between energy losses and the available temperature difference, providing an evaluation of energy conversion under the given temperature-difference conditions available. Exergy efficiency pays more attention to the sustainability and effectiveness of energy utilization, reflecting the degree of the device’s utilization of low-grade energy. The combined use of thermal efficiency and exergy efficiency provides a comprehensive understanding of the performance of the thermoelectric power generation device.

#### 2.3.1. Thermal Efficiency

The thermal efficiency η of the system is as follows:

(8) $$\eta =\frac{P}{Q_{h}}=\frac{I^{2}R}{\alpha T_{h}I+k\left(T_{h}-T_{c}\right)-\frac{1}{2}\cdot I^{2}R_{0}}$$

where *P*—output power; $Q_{h}$—heat absorbed from heat source; *R*—external resistance; *I*—current; α—Seebeck coefficient; $T_{h}$—hot end temperature, $T_{c}$—cold end temperature; k—thermal conductivity; $R_{0}$—internal resistance.

#### 2.3.2. Exergy Efficiency

The exergy efficiency $\eta_{x}$ of the system is as follows:

(9) $$\eta_{x}=\frac{E_{x}}{E_{xQ}}=\frac{I^{2}R}{\left(1-\frac{T_{c}}{T_{h}}\right)Q_{h}}=\frac{\eta }{1-\frac{T_{c}}{T_{h}}}$$

$\mathrm{where}\;E_{x}—$income exergy; $E_{xQ}—$payout exergy; $\eta_{x}—$exergy efficiency.

## 3. Experimental Results and Analysis

### Performance Enhancement of the System

The cable-surface-waste-heat-recovery thermoelectric power generation device is a system that utilizes the Seebeck effect to convert waste heat on the surface of a cable into electrical energy. To enhance the performance of this system, fins can be used to improve the heat transfer between the hot and cold sides of the thermoelectric power generation device, as shown in Figure 1a. The number of teeth on the fins is a key factor that influences the thermoelectric conversion performance. In this section of the discussion, we will investigate the influence of the number of teeth on the output power of the thermoelectric power generation device. For comparison purposes, we installed six different types of cooling fins (Cases 1–6), while the device without cooling fins served as the baseline and is labeled Case 0.

**Figure 2:**
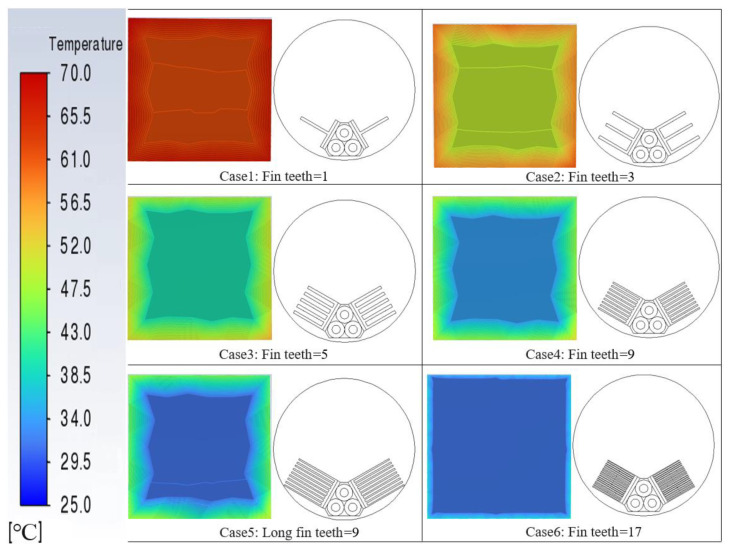
Cold-side temperature distribution and different types of fins.

Figure 2 presents a temperature distribution cloud map of the cold-side surface and a two-dimensional diagram of the fins for the cable-surface-waste-heat-recovery thermoelectric power generation device. As can be seen from the figure, with an increase in the number of fin teeth, the temperature of the cold-side surface gradually decreases. When the number of fin teeth reaches 17 (Case 6), the temperature becomes highly uniform on the cold-side surface and reaches its lowest temperature. This is because installing multi-toothed fins can increase the heat transfer surface area, enhance heat transfer efficiency, and improve fluid turbulence and heat transfer effectiveness. With an increased number of fin teeth, a larger heat transfer surface area is provided, resulting in enhanced heat dissipation and a lowered surface temperature. Furthermore, a smaller spacing between the multi-toothed fins can better disrupt the fluid flow and enhance the turbulent effect, further improving the heat transfer efficiency and achieving a more uniform temperature distribution on the surface. These research findings indicate that the cable-surface-waste-heat-recovery thermoelectric power generation device with 17-tooth fins (Case 6) on its cold side exhibits the highest maximum output power and heat transfer coefficient within the pipe. This is attributed to the larger heat-transfer surface area of the 17-tooth fin compared to other types of fins, enabling a more effective heat transfer capacity, improved heat transfer efficiency, a reduction in the temperature on the cold side of the thermoelectric power generation device, an increased temperature difference between the hot and cold sides, and, consequently, increased output power. Based on the installation of fins with different numbers of teeth, the maximum output power can be increased by nearly 30 times.

**Figure 3:**
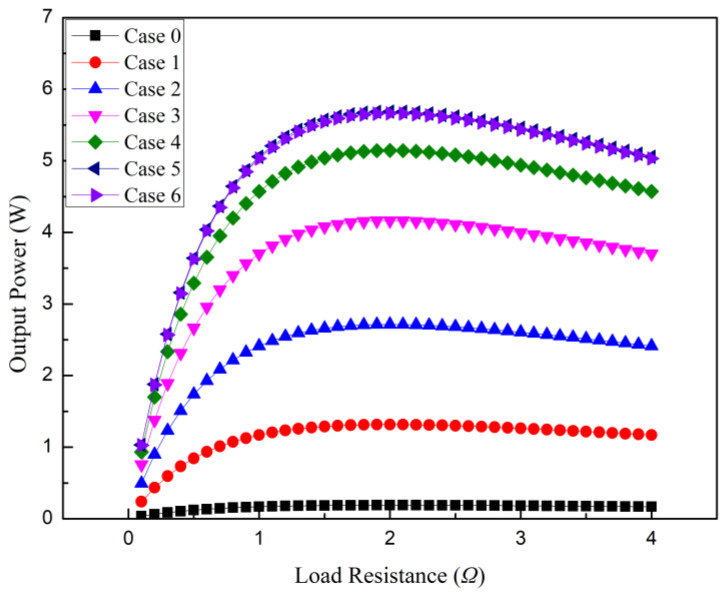
System output power for different Cases.

Figure 3 shows the variation in the output power of the cable-surface-waste-heat-recovery thermoelectric power generation device with the external load resistance. This pattern plays a crucial role in understanding and optimizing the performance of the thermoelectric power generation device. The phenomenon can be explained as follows: firstly, when the external load resistance is zero, the entire system is in a short-circuit state, and no current can flow in the system, resulting in zero output power. In this case, there is no resistance within the circuit to impede the flow of the current, leading to a lack of output power generation. However, as the external load resistance increases, the current in the system circuit gradually increases, resulting in the generation of more electrical energy and an increase in the output power. The increase in the external load resistance leads to an increase in the current within the system circuit, allowing the thermoelectric power generation device to more fully convert thermal energy into electrical energy output. Therefore, as the external load resistance increases, the output power of the entire system also increases. When the external load resistance gradually approaches the internal total resistance of the cable-surface-waste-heat-recovery thermoelectric power generation device, the system circuit reaches its maximum power transmission state, and the output power reaches its peak. At this point, the internal resistance of the system’s circuit matches the external load resistance perfectly, maximizing the current flow. This means that the thermoelectric power generation device can convert thermal energy into electrical energy output at its highest efficiency, resulting in maximum output power. However, if the external load resistance continues to increase beyond this point, the current within the system circuit gradually decreases, and the output power begins to decline. This is because surpassing the maximum power transmission state reduces the power transmission efficiency within the circuit. The increase in external load resistance leads to an increase in the total resistance within the circuit, limiting the flow of current and resulting in less thermal energy being converted into output power. Hence, the output power gradually decreases. In summary, there exists an optimal matching state between the external load resistance and the output power. When the external load resistance matches the internal total resistance of the thermoelectric power generation device, the system’s circuit reaches its maximum power transmission state, and the output power reaches its maximum value. If the system’s circuit exceeds this matching state, further increases in the external load resistance will cause a decrease in the current and, consequently, a reduction in the output power. Therefore, understanding and optimizing the relationship between the external load resistance and the output power are crucial for improving the performance of the cable-surface-waste-heat-recovery thermoelectric power generation device.

**Figure 4:**
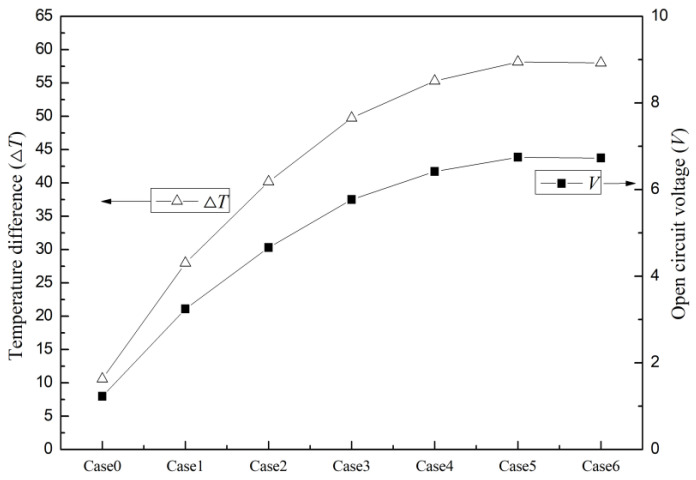
System temperature difference and open circuit voltage for different Cases.

Based on the research results, it can be observed that the cable-surface-waste-heat-recovery thermoelectric power generation device equipped with cooling fins (Case 1–6) exhibits a higher level of output power compared to the device without cooling fins (Case 0). Among the configurations with cooling fins, the device with 17-tooth cooling fins (Case 6) demonstrates the highest output power, while the device with single-tooth cooling fins (Case 1) exhibits the lowest output power. This can be attributed to the improved heat dissipation efficiency on the cold side of the thermoelectric power generation device when cooling fins are installed. The design of the cooling fins allows for the faster conduction of heat from the cold-side surface to the fins, facilitating heat dissipation and reducing the temperature. Therefore, the installation of cooling fins effectively lowers the temperature on the cold side of the thermoelectric modules on the cable surface, providing a larger temperature difference and increasing the thermal efficiency of the thermoelectric power generation device. Additionally, the presence of cooling fins expands the heat exchange surface area on the cold side of the thermoelectric power generation device. The installation of fins increases the area of contact with the ambient low-temperature air, making more surface area available for absorbing coldness from the environment. As a result, the thermoelectric power generation device can more fully utilize the temperature difference between the hot and cold sides, facilitating more efficient heat transfer and consequently improving the output power. Furthermore, different designs of cooling fins also have an impact on the output power. As shown in the figure, the device with 17-tooth cooling fins exhibits the highest output power, while the device with single-tooth cooling fins shows the lowest output power. This is because the design of the 17-tooth cooling fins induces a gas flow disturbance when the low-temperature air passes through the fins. With more fins present, the air flow experiences more redirection and flow changes between the fins. This intensified gas flow disturbance increases the contact area between the gas and the fin surface, enhancing heat conduction and heat transfer. By increasing the contact area between the gas and the fins, the 17-tooth cooling fins can more effectively absorb coldness from their surroundings and transfer it to the thermoelectric power generation device. In contrast, the single-tooth cooling fins have fewer fins, resulting in less gas flow disturbance and a relatively smaller contact area, thereby leading to a weaker heat conduction capacity. In summary, the intensified gas flow disturbance caused by the 17-tooth cooling fins has a positive effect on the output power of the thermoelectric power generation device. It provides more contact area and enhances heat conduction, thereby increasing the heat transfer from the cold side and subsequently raising the temperature difference and open circuit voltage of the device. On the other hand, as the number of teeth on the cooling fins increases, the temperature difference and open circuit voltage of the device also increase, as shown in Figure 4. However, it can be observed from Figure 3 and Figure 4 that the influences of the 9-tooth long cooling fins and the 17-tooth cooling fins on the performance of the device are similar. This similarity arises from the fact that the increased gap length in the 9-tooth long cooling fins and the increased number of gaps in the 17-tooth cooling fins provide similar levels of gas flow disturbance within the gaps. Similarly, the increased tooth length in the 9-tooth long cooling fins and the increased number of teeth in the 17-tooth cooling fins offer comparable expanded contact areas, resulting in similar levels of heat exchange with the surrounding cold air. The combined effect of these two factors leads to similar results in terms of the temperature difference between the hot and cold sides of the thermoelectric modules, open circuit voltage, and output power for Case 5 and Case 6.

**Figure 5:**
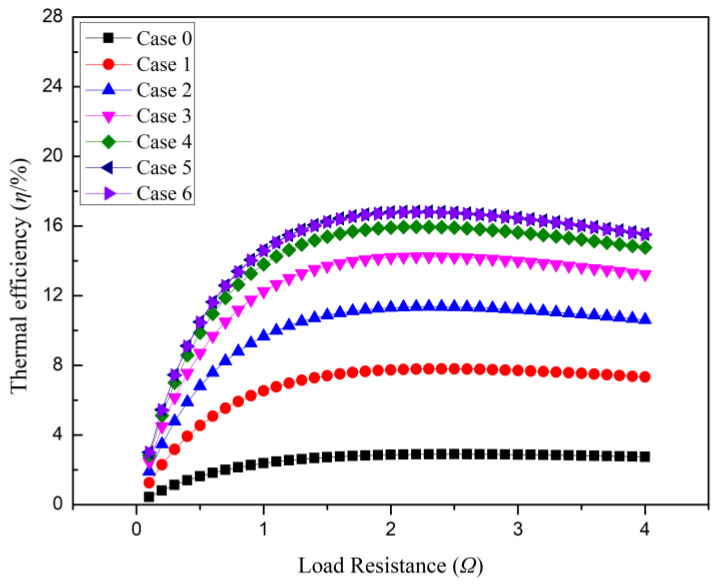
System thermal efficiency.

**Figure 6:**
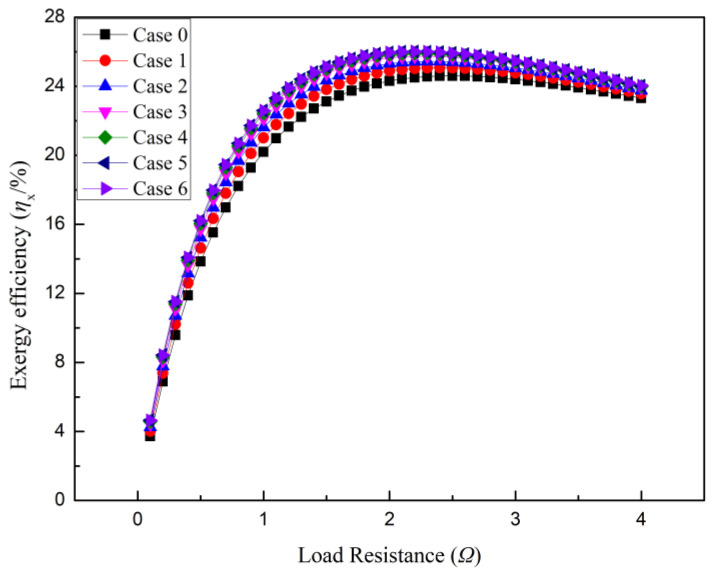
System exergy efficiency.

Figure 5 and Figure 6 illustrate the variations in the thermal efficiency and exergy efficiency of the cable-surface-waste-heat-recovery thermoelectric power generation device with different types of cooling fins as the external resistance changes. As shown in the figures, both the thermal efficiency and exergy efficiency exhibit a peak value as the external resistance gradually increases from zero, and these peak values occur when the external resistance equals the internal resistance of the thermoelectric power generation device. By comparing the two efficiency measures, it can be observed that as the external resistance changes, the exergy efficiency increases more rapidly than the thermal efficiency. This indicates that the exergy efficiency of the system is relatively sensitive to variations in the external resistance of the thermoelectric power generation device, particularly around the peak value. However, beyond the peak value, as the external resistance continues to increase, both the exergy efficiency and thermal efficiency exhibit relatively gentle decreasing trends, although the decline in exergy efficiency is slightly more noticeable. This phenomenon can be attributed to the fact that the thermal efficiency of the system represents the ratio between the converted thermal energy output of the cable-surface-waste-heat-recovery thermoelectric power generation device and the supplied thermal energy, i.e., the ratio of the output power to the input thermal power. On the other hand, the exergy efficiency refers to the ratio between the converted thermal energy output of the thermoelectric power generation device and the available temperature difference, i.e., the ratio of the output power to the available temperature difference. When the external resistance equals the total internal resistance of the thermoelectric power generation device, a match between the internal and external resistances is achieved. In this case, the thermoelectric power generation device can fully utilize the energy in the external circuit, resulting in improved thermal energy conversion efficiency and thus reaching peak thermal efficiency and exergy efficiency. As the external resistance changes, the exergy efficiency is more sensitive to variations in the external resistance compared to the thermal efficiency. The increase in exergy efficiency primarily relies on the utilization efficiency of the available temperature difference and the thermal energy output converted by the system, and variations in external resistance can significantly affect the convertible thermal energy output. Therefore, when the external resistance approaches the internal resistance, even minor changes in resistance can lead to a rapid increase in the exergy efficiency. As the external resistance continues to increase, the overall system resistance increases, which affects the thermoelectric conversion capability of the cable-surface-waste-heat-recovery thermoelectric power generation device. When the external resistance exceeds the total internal resistance of the system, the thermoelectric conversion capability between the cable and the thermoelectric power generation device weakens, resulting in a decline in both the thermal efficiency and exergy efficiency. However, the exergy efficiency is relatively more sensitive because it depends more on the effective conversion and output of thermal energy. Therefore, the cable-surface-waste-heat-recovery thermoelectric power generation device exhibits an increasing trend, followed by a decreasing trend for both thermal efficiency and exergy efficiency as the external resistance changes. This can be attributed to the matching of internal resistance, the sensitivity of the exergy efficiency, and the impact of an increase in resistance on the thermoelectric conversion.

It can also be observed from the figures that the thermal efficiency and exergy efficiency of the cable-surface-waste-heat-recovery thermoelectric power generation device are higher when cooling fins are installed compared to when they are not installed. Furthermore, the thermal efficiency and exergy efficiency of the thermoelectric power generation device with 9-long-tooth fins (Case 5) and 17-tooth fins (Case 6) are higher than those with other types of fins, and the initial increasing trend of the exergy efficiency is more significant than that of the thermal efficiency. The reason for this result is that the installation of cooling fins increases the heat dissipation surface area on the cold side of the thermoelectric power generation device, providing more surface area for heat exchange and enhancing heat conduction and absorption. This facilitates a more efficient utilization of the temperature difference resource and improves both the thermal efficiency and exergy efficiency. Cooling fins also improve the conduction of heat within the thermoelectric power generation device. By increasing the number and length of fins, the conduction path of the heat within the device becomes more extensive, enhancing the device’s heat conduction capability. This helps to increase the conduction of heat from the cold side to the fins, thereby improving the thermal efficiency and exergy efficiency of the system. Similarly, cooling fins enhance heat dissipation by increasing the surface area and surface structure. More fins provide a larger surface area for heat dissipation, facilitating the rapid dissipation of heat into the surrounding environment. This reduces the increase in temperature on the cold side of the device, maintains a higher temperature difference, and improves the thermal efficiency and exergy efficiency of the system. The thermoelectric power generation devices with 9-long-tooth fins (Case 5) and 17-tooth fins (Case 6) can more effectively utilize the available temperature difference resource. The designs of these fins are better-suited for the current temperature difference conditions, enabling them to capture and utilize the temperature difference resource more effectively, thereby improving the thermal efficiency and exergy efficiency. The more pronounced initial increasing trend of the exergy efficiency compared to the thermal efficiency when the external resistance increases is due to the fact that at lower external resistances, the thermoelectric power generation device can more fully utilize the available temperature difference resource to enhance the intensity of thermal energy conversion and improve the system’s exergy efficiency. As the external resistance increases beyond the total internal resistance of the system, the gain in convertible thermal energy output diminishes, resulting in a relatively flattened growth trend for the exergy efficiency.

**Figure 7:**
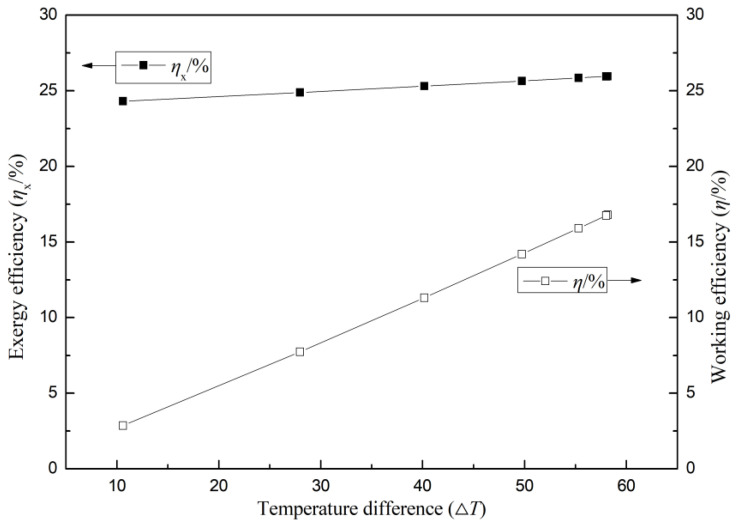
System thermal efficiency and exergy efficiency under different temperature differences.

Figure 7 shows the variation in the thermal efficiency and exergy efficiency of the cable-surface-waste-heat-recovery thermoelectric power generation device with the temperature difference between the hot and cold sides. It can be observed from the figure that both the thermal efficiency and exergy efficiency of the thermoelectric power generation device increase with the increase in the temperature difference, but the increase in thermal efficiency is more significant than the increase in exergy efficiency. This is because the installation of cooling fins increases the heat conduction capability on the cold side of the thermoelectric module and also introduces some level of flow disturbance into the gas flow in the pipe. The enhanced gas flow disturbance further improves the heat dissipation effect of the fins, resulting in a reduction in the temperature difference on the cold side of the thermoelectric power generation device. Additionally, the cooling fins have a larger heat transfer area, which effectively assists in transferring heat within the system. This allows the cold side of the thermoelectric module attached to the cable surface to more fully utilize the heat transfer provided by the fins, thereby generating a larger temperature difference and higher thermal and exergy efficiencies. Furthermore, this phenomenon reaches an optimal state when using 9-long-tooth fins and 17-tooth fins in the thermoelectric power generation device. The conduction of heat within the thermoelectric power generation device is a critical factor in achieving energy conversion. The efficiency of heat conduction is influenced by several factors, including the properties of the heat conduction medium, the characteristics of the thermoelectric module, and the heat transfer paths within the system. As the temperature difference between the hot and cold sides of the thermoelectric power generation device increases, the intensity of heat conduction within the system also increases, thereby improving the device’s thermal efficiency. However, when considering efficiency, the heat conduction losses must be taken into account. This means that the improvement in the exergy efficiency is limited by energy losses during the heat conduction process. The characteristics of the thermoelectric module also play a crucial role in the performance of the thermoelectric power generation device. As the temperature difference between the hot and cold sides increases, high-quality thermoelectric modules exhibit better thermoelectric performance, i.e., higher thermal-to-electric conversion efficiencies, thus improving the thermal efficiency of the thermoelectric power generation device. However, the characteristics of the thermoelectric module, such as the thermal conductivity and electrical conductivity, also influence the system’s performance and affect the rate of increase in exergy efficiency. Exergy efficiency is an efficiency indicator that considers energy losses and is the ratio between the thermal efficiency and energy conduction losses, as shown in equation 9. Therefore, as the temperature difference between the hot and cold sides increases, the energy losses within the system also increase, resulting in a relatively smaller increase in exergy efficiency. This is because exergy efficiency takes into account the impact of energy losses, and its increase is constrained by energy losses. In contrast, thermal efficiency only considers the proportion of thermal energy converted into electrical energy and does not directly consider energy losses. Therefore, as the temperature difference increases, the increase in thermal efficiency becomes more significant, while the increase in exergy efficiency is limited and becomes slower.

## 4. Conclusions

This study investigated the thermal efficiency and exergy efficiency of a cable-surface-waste-heat-recovery thermoelectric power generation device and examined the impact of using different types of cooling fins on the system’s performance through numerical simulations. The following conclusions can be drawn from the analysis and research results:

1. The installation of cooling fins on the thermoelectric power generation device improves the performance by enhancing heat transfer and heat dissipation. With an increased number of fin teeth, the temperature on the cold-side surface becomes more uniform and reaches its lowest value. The device with 17-tooth fins exhibited the highest maximum output power and heat transfer coefficient due to its larger heat transfer surface area, improved heat transfer efficiency, and reduced temperature on the cold side.
2. The thermoelectric power generation device demonstrates an increase in its output power with an increase in the external load resistance, reaching its peak value at a certain threshold. However, beyond this critical point, the output power starts to decline. Therefore, achieving the optimal matching state between the external load resistance and the output power becomes crucial for maximizing the device’s overall efficiency.
3. The thermal efficiency and exergy efficiency of the thermoelectric power generation device are higher when cooling fins are installed compared to when they are not installed. The devices with 9-long-tooth fins and 17-tooth fins demonstrated higher efficiencies compared to other types of fins. The initial increasing trend of the exergy efficiency is more significant than that of the thermal efficiency, indicating the sensitivity of exergy efficiency to variations in the external resistance of the device.
4. Both the thermal efficiency and exergy efficiency of the thermoelectric power generation device increase with an increase in the temperature difference between the hot and cold sides. However, the increase in the thermal efficiency is more significant than the increase in the exergy efficiency. The improvement in thermal efficiency is influenced by factors such as enhanced heat conduction and the properties of the thermoelectric module, while the increase in exergy efficiency is limited by energy losses during the heat conduction process.

Overall, the installation of cooling fins and the optimization of external load resistance and temperature difference can significantly enhance the performance of a cable-surface-waste-heat-recovery thermoelectric power generation device. The combined use of thermal efficiency and exergy efficiency provides a comprehensive evaluation of the device’s performance from different perspectives, considering its energy conversion efficiency and the utilization of available temperature-difference resources.

## Data Availability

All data used are reported herein and/or in the cited literature sources.

## References

[1] Zhao Y., Wang S., Ge M., Li Y., Liang Z., Yang Y. (2018). Performance analysis of a thermoelectric generator applied to wet flue gas waste heat recovery. Appl. Energy.

[2] Zhao Y., Fan Y., Li W., Li Y., Ge M., Xie L. (2022). Experimental investigation of heat pipe thermoelectric generator. Energy Convers. Manag..

[3] Zhao Y., Lu M., Li Y., Ge M., Xie L., Liu L. (2021). Characteristics analysis of an exhaust thermoelectric generator system with heat transfer fluid circulation. Appl. Energy.

[4] Fu Y., Li Y. (2023). Experimental Study on the Working Efficiency and Exergy Efficiency of the Vehicle-Mounted Thermoelectric Generator for Cold Chain Logistics Transportation Vehicle. Processes.

[5] Li Y., Wang S., Zhao Y., Lu C. (2017). Experimental study on the influence of porous foam metal filled in the core flow region on the performance of thermoelectric generators. Appl. Energy.

[6] Li Y., Wang S., Zhao Y., Yue L. (2022). Effect of thermoelectric modules with different characteristics on the performance of thermoelectric generators inserted in the central flow region with porous foam copper. Appl. Energy.

[7] Li Y., Wang S., Zhao Y., Yue L. (2022). Experimental study on the effect of core flow heat transfer enhancement on the performance of TEG. Energy Rep..

[8] He W., Wang S., Lu C., Zhang X., Li Y. (2016). Influence of different cooling methods on thermoelectric performance of an engine exhaust gas waste heat recovery system. Appl. Energy.

[9] He W., Wang S., Zhang X., Li Y., Lu C. (2015). Optimization design method of thermoelectric generator based on exhaust gas parameters for recovery of engine waste heat. Energy.

[10] Zhao Y., Lu M., Li Y., Wang Y., Ge M. (2023). Numerical investigation of an exhaust thermoelectric generator with a perforated plate. Energy.

[11] Zhao Y., Wang S., Ge M., Liang Z., Liang Y., Li Y. (2018). Performance analysis of automobile exhaust thermoelectric generator system with media fluid. Energy Convers. Manag..

[12] Zhao Y., Wang S., Ge M., Liang Z., Liang Y., Li Y. (2019). Performance investigation of an intermediate fluid thermoelectric generator for automobile exhaust waste heat recovery. Appl. Energy.

[13] Ge M., Zhao Y., Li Y., He W., Xie L., Zhao Y. (2022). Structural optimization of thermoelectric modules in a concentration photovoltaic–thermoelectric hybrid system. Energy.

[14] Ge M., Li Z., Zhao Y., Xuan Z., Li Y., Zhao Y. (2022). Experimental study of thermoelectric generator with different numbers of modules for waste heat recovery. Appl. Energy.

[15] Luo D., Yan Y., Chen W., Yang X., Chen H., Cao B., Zhao Y. (2023). A comprehensive hybrid transient CFD-thermal resistance model for automobile thermoelectric generators. Int. J. Heat Mass Transf..

[16] Li Y., Wang S., Fu Y., Zhao Y., Yue L. (2022). Effect of core flow heat transfer enhancement on power generation characteristics of thermoelectric generators with different performances. Therm. Sci..

[17] Li Y., Wang S., Fu Y., Zhao Y., Yue L. (2022). Influence of foamed metal core flow heat transfer enhancement on the performance of thermoelectric generators with different power generation characteristics. Therm. Sci. Eng. Prog..

[18] Li Y., Wang S., Zhao Y. (2018). Experimental study on heat transfer enhancement of gas tube partially filled with metal foam. Exp. Therm. Fluid Sci..

[19] Wang C.C., Hung C.I., Chen W.H. (2012). Design of heat sink for improving the performance of thermoelectric generator using two-stage optimization. Energy.

[20] Liu J., Yadav S., Kim S.C. (2022). Performance of a thermoelectric generator system for waste heat recovery utilizing plate fin heat sink in bronze ingot casting industry. Case Stud. Therm. Eng..

[21] Seo Y.M., Ha M.Y., Park S.H., Lee G.H., Kim Y.S., Park Y.G. (2018). A numerical study on the performance of the thermoelectric module with different heat sink shapes. Appl. Therm. Eng..

[22] Pujol T., T’Jollyn I., Massaguer E., Massaguer A., Cózar I.R., De Paepe M. (2023). Design optimization of plate-fin heat sink with forced convection for single-module thermoelectric generator. Appl. Therm. Eng..

[23] Kang Y.K., Joung J., Kim M., Jeong J.W. (2023). Energy impact of heat pipe-assisted microencapsulated phase change material heat sink for photovoltaic and thermoelectric generator hybrid panel. Renew. Energy.

[24] Zheng L.J., Kang H.W. (2022). A passive evaporative cooling heat sink method for enhancing low-grade waste heat recovery capacity of thermoelectric generators. Energy Convers. Manag..

[25] Lee H.S. (2022). Thermal Design: Heat Sinks, Thermoelectrics, Heat Pipes, Compact Heat Exchangers, and Solar Cells.

[26] Jang J.Y., Tsai Y.C., Wu C.W. (2013). A study of 3-D numerical simulation and comparison with experimental results on turbulent flow of venting flue gas using thermoelectric generator modules and plate fin heat sink. Energy.

[27] Ma T., Lu X., Pandit J., Ekkad S.V., Huxtable S.T., Deshpande S., Wang Q.W. (2017). Numerical study on thermoelectric–hydraulic performance of a thermoelectric power generator with a plate-fin heat exchanger with longitudinal vortex generators. Appl. Energy.

[28] Naphon P., Wiriyasart S. (2009). Liquid cooling in the mini-rectangular fin heat sink with and without thermoelectric for CPU. Int. Commun. Heat Mass Transf..

[29] Wang Y., Wu C., Tang Z., Yang X., Deng Y., Su C. (2015). Optimization of fin distribution to improve the temperature uniformity of a heat exchanger in a thermoelectric generator. J. Electron. Mater..

[30] Chen W.H., Chiou Y.B., Chein R.Y., Uan J.Y., Wang X.D. (2022). Power generation of thermoelectric generator with plate fins for recovering low-temperature waste heat. Appl. Energy.

[31] Wang R., Fu Y., Luo D., Chen J., Zhou W. (2022). Performance evaluation of an automotive thermoelectric generator with a non-isometric distributed fin heat exchanger. Sustain. Energy Fuels.

[32] Xu Y., Islam M.D., Kharoua N. (2017). Numerical study of winglets vortex generator effects on thermal performance in a circular pipe. Int. J. Therm. Sci..

